# Supplementary material for: Modulating the Heat Sensitivity of Prostate Cancer Cell Lines In Vitro: A New Impact for Focal Therapies
Source: Biomedicines. 2020 Dec 9;8(12):585. doi: 10.3390/biomedicines8120585 (PMC7763367; doi:10.3390/biomedicines8120585)
Supplement: Supplementary file 1 [file biomedicines-08-00585-s001.zip › suppl_checked.docx]

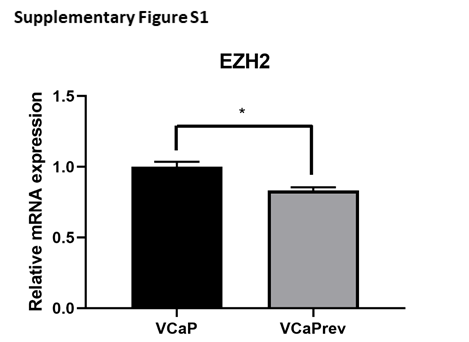


**Figure S1.** Gene expression of EZH2 in VCaP and VCaPrev cells. Results are mean ± SEM (*n* = 3). Significance was calculated with unpaired *t*-test, * *p* < 0.05.
